# Codeveloping an Adolescent Health Program in India During the COVID-19 Pandemic: A Case Study of a Community-Partnered Responsive Feedback Approach

**DOI:** 10.9745/GHSP-D-22-00224

**Published:** 2023-12-18

**Authors:** Ricky Sharma, Mohammed Danish, Yasmin Khajenoori, Daniya Shaikh, Yasmeen Chaudary, Hiba Khan, Neha Bhat, Avani Doshi, Samruddhi Nalawade, Pawan Rajput, Snigdha Shahi, Priya Shankar

**Affiliations:** a Adolescent Health Champions, San Francisco, CA, USA.; b Adolescent Health Champions, Delhi, India.; c University of California, San Francisco, San Francisco, CA, USA.; d Adolescent Health Champions, Mumbai, India.

## Abstract

We describe the responsive feedback (RF) approach experience of a nongovernmental organization, Girls Health Champions (now known as Adolescent Health Champions [AHC]), that undertakes peer education interventions in Mumbai, India, schools to improve gender equality and health outcomes for adolescents aged 13–19 years. AHC used the RF approach at the onset of the COVID-19 pandemic in light of uncertainties stemming from school closures and the negative impact of the lockdown on adolescents' physical and mental health. Using an RF approach, AHC was able to: (1) understand pandemic-specific challenges faced by adolescents; (2) overhaul its theory of change; (3) pilot new modes of intervention delivery; (4) design a curriculum for parents/guardians and a COVID-19 module; (5) design an AHC mobile app; (6) develop a new, more gender-inclusive name and visual identity; (7) change the overall structure, adolescent-friendly nature, and agility of the organization; (8) and help clarify future directions taken by the organization. Overall, use of the RF approach had significant positive impacts on AHC as an organization, such as changes in organizational culture, deeper stakeholder engagement, and innovation, and was instrumental in AHC's growth, development, and pandemic response. This article outlines the steps of the process, from initial informal stakeholder consultations to the eventual formalization of the RF approach into the everyday working of AHC through the creation of a youth advisory board. We discuss challenges, such as time and resource constraints encountered; strategies for dealing with such challenges; and general key findings and learnings from this experience that could be beneficial to other youth- and community-serving organizations.

## Abstract (precis)

Adolescent Health Champions used responsive feedback to adapt its peer education intervention to address negative impacts on adolescents' mental and physical health and closures caused by the COVID-19 pandemic.

## Teaser

**Key Findings:**

- The responsive feedback (RF) approach was an effective way for Adolescent Health Champions to bolster adolescent and community stakeholder engagement by creating a youth advisory board to address COVID-19-related challenges to an in-person peer education intervention.
- Using RF helped Adolescent Health Champions and the youth advisory board to collaboratively use design thinking to understand pandemic-specific adolescent challenges, pilot new modes of intervention delivery, develop a mobile app for adolescent health, and develop a more gender-inclusive name and visual identity.
- Adolescent Health Champions shifted from using traditional metrics (e.g., number of adolescents educated) to using a broader vision centered on its ability to innovate, increase adolescent inclusivity in all aspects of the organization and decision-making, and adapt to situations as needs arose.

**Key Implications:**

- Adolescent-serving organizations can strengthen engagement with adolescent and community stakeholders by adopting the RF approach.
- Formalizing the RF approach into the everyday workings of an organization can lead to multiple qualitative changes in organizational culture, a broader vision of success, deeper and more regular stakeholder engagement, and the adoption of innovative approaches.

## BACKGROUND

India is home to the world's largest adolescent population, with more than 253 million youth aged 10–19 years.^1^ The rapid mental, physical, and social changes that occur during this life stage can prove to be challenging for adolescents. Additionally, adolescents in India face other issues, such as inadequate health education, a lack of adolescent-friendly health care resources, and deeply entrenched gender inequality. These challenges are reflected in the health issues that India's adolescents, particularly girls, experience. Approximately 50% of adolescent girls aged 15–19 years are anemic—twice the rate for boys. Girls also face a nearly 30% likelihood of sexual abuse and a roughly 20% teen pregnancy rate.^2^ Mumbai district-level data suggests that 1 in 10 women are married before they are aged 18 years.^3^

Studies further indicate a high prevalence of mental illness among adolescents.^4^ For example, mental health issues among adolescents in Mumbai are a challenge, with 1 study showing that 40% of sampled girls reported thoughts that they would be better off dead or hurting themselves, and 14.8% of adolescents of all genders described psychiatric morbidity.^5^ An additional study found that more than 30% of girls reported unsatisfactory menstrual hygiene practices, and only 19% of adolescents knew about safe sexual practices.^6^ The COVID-19 pandemic has further impacted the mental and physical health of adolescents, exacerbating many of the adverse health outcomes that adolescents had been experiencing before the pandemic. India saw a sharp rise in anxiety, depression, and physical health issues, such as weight gain and decreased physical fitness, among adolescents.^7^

To help adolescents respond to these challenges, in 2016, Adolescent Health Champions (AHC, formerly known as Girls Health Champions) began working in schools in Mumbai and Delhi to deliver an in-person school-based peer education intervention to adolescents aged 13–19 years. Recent global meta-reviews of peer education interventions have shown mixed results on peer education's effectiveness and highlighted common shortcomings in its implementation.^8^^,^^9^ To better understand the conditions under which peer education is successful and to what degree it can affect knowledge, attitudes, and behavior changes, more data from intentional and well-designed interventions based on peer education is needed. A key factor in the success and effectiveness of these approaches could be stakeholder engagement, in this case, engagement with adolescents.

In 2020, after COVID-19 forced lockdowns and school closures in India, AHC wanted to formulate alternative strategies in response to school closures that disrupted in-person programming and adapted to changing adolescent health needs during the pandemic. To that end, AHC created a youth advisory board (YAB) in 2020 as part of a responsive feedback (RF) approach to involve adolescent stakeholders in decision-making and address changing adolescent health needs during the pandemic.

AHC used an RF approach to formulate alternative strategies in response to school closures that disrupted in-person programming and to better adapt to changing adolescent health needs during the pandemic.

AHC's decision to use an RF approach further takes cues from present gaps in literature around peer education and the growing international consensus around the importance of meaningful adolescent engagement and leadership in adolescent-serving organizations.^10^^,^^11^ This article describes the process and key organizational learnings from following this adolescent-focused RF approach.

**FIGURE 1:**
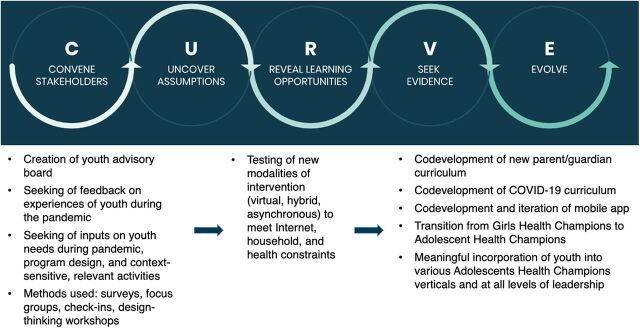
Overview of Responsive Feedback Approach Used by the Adolescent Health Champions Program

A YAB is a body of young people appointed by an organization to inform and advise policies and practices that directly impact the youth community.^12^ YABs, by virtue of their purpose and composition, are a key means to close the gap between a program and the adolescents it hopes to serve, ensure seamless and timely communication, bolster youth and adolescent engagement and participation, and foster joint reflection sessions on the effectiveness of an intervention or activity in achieving its and the organization's goals. AHC views RF as a platform and approach that successfully builds strong stakeholder relationships such that a space for reflection, testing, and learning with the adolescents could be cultivated and sustainably maintained (Figure 1).^13^ RF additionally incorporates elements, such as being agile, adaptable, iterative, responsive, and actionable.

## AHC INTERVENTION

Co-founded in 2016 by Ricky Sharma and Priya Shankar, Girls Health Champions, now known as Adolescent Health Champions, is a U.S.-based organization that delivers a school-based peer health education intervention alongside partner Indian nongovernmental organizations to adolescents aged 13–19 years and studying in 8th, 9th, and 10th standards (grades) in Indian private and government-aided schools. In 2020, the AHC team had less than 10 members, 80% of whom were aged younger than 25 years. Being a relatively small team comprised mainly of young adults was an essential characteristic, as it helped AHC be closer to the lived experiences of adolescents. AHC addresses gender inequality and the leading causes of adolescent morbidity and mortality. The AHC health education curriculum is divided into 4 distinct educational modules: (1) nutrition and anemia, (2) mental health and substance abuse, (3) gender and relationships, and (4) sexual and reproductive health (Table 1). These modules were developed in collaboration with pediatricians, obstetricians/gynecologists, psychiatrists, psychologists, adolescents, and educational experts from India and the United States. The modules also closely follow the pedagogical guidelines laid out in India's 2014 national health policy for adolescents, Rashtriya Kishor Swasthya Karyakram (RKSK), which focuses on nutrition, reproductive health, and substance abuse, among other issues. The AHC curriculum is meant to be taught in parallel with the RKSK and complement and add to the RKSK syllabus wherever required. Of note, while the RKSK program works in mainly government and out-of-school settings, the AHC curriculum is a school-based program that initially has been piloted in government-aided and private schools.

**TABLE 1.** Adolescent Health Champions Modular Content

Module Name	Content
Nutrition and anemia	Balanced diets, iron deficiency anemia, ways to prevent iron deficiency anemia and undernutrition, gender differences and subsequent nutritional deficiencies
Mental health and substance abuse	Mental health issues (stress, emotions, mind and body connection, depression, anxiety, and other mental illnesses), self-care and self-love, self-esteem, body image, coping strategies, seeking mental health support, addiction
Gender and relationships	Gender identity and expression, sex assigned at birth, sexual orientation, toxic masculinity, gender roles, gender, caste or religious-based violence, child marriage, healthy relationships, safe and unsafe touch, assault and staying safe
Reproductive health	Reproductive anatomy, menstruation, menstrual taboos, menstrual irregularities, sexual intercourse, pregnancy, pregnancy prevention, HIV/AIDS, other sexually transmitted infections, contraception, abortion, family planning

The AHC intervention involves training a subset of adolescents in each school as peer health educators, known as champions. Each group of champions receives 4–6 hours of training with the AHC team, during which they practice teaching 1 thematic module of their choice. Champions are selected on a voluntary basis from the larger student population attending government-aided and private schools in Mumbai. During champion selection, if the number of volunteers exceeds the number of peer educator slots, then champions are selected at random by drawing lots. Teachers are consulted only after the peer educators have been selected to confirm if any of the selected peer educators might need additional support during training (such as individuals with learning disabilities).

The AHC intervention involves training a subset of adolescents in each school as peer health educators, known as champions.

After they undergo initial peer education training, the champions are provided structured opportunities within schools to teach the AHC curriculum to their classmates. The champions spend approximately 2 hours teaching the content and 30–60 minutes facilitating activities and answering questions about each module. Session timings are tailored to each school's schedule, and the AHC team remains on standby for support during these sessions. In each school, copies of the AHC curriculum are given to the champions, teachers, and students, and AHC returns 2–3 times a year for refresher training led by these champions to review curricular content. At the end of each year, new champions are trained to ensure continuity. Thus, AHC ensures quality peer education by: (1) investing in training champions, (2) being available to support peer educators and answer any technical questions during the sessions, and (3) engaging with schools at multiple touchpoints over the academic year. Since its inception, AHC has aimed to amplify community and, especially, adolescent voices to guide and strengthen the intervention. This proved to be particularly important during the COVID-19 pandemic, a rapidly evolving and unpredictable time when school closures disrupted AHC's in-person peer education model.

**FIGURE 2:**
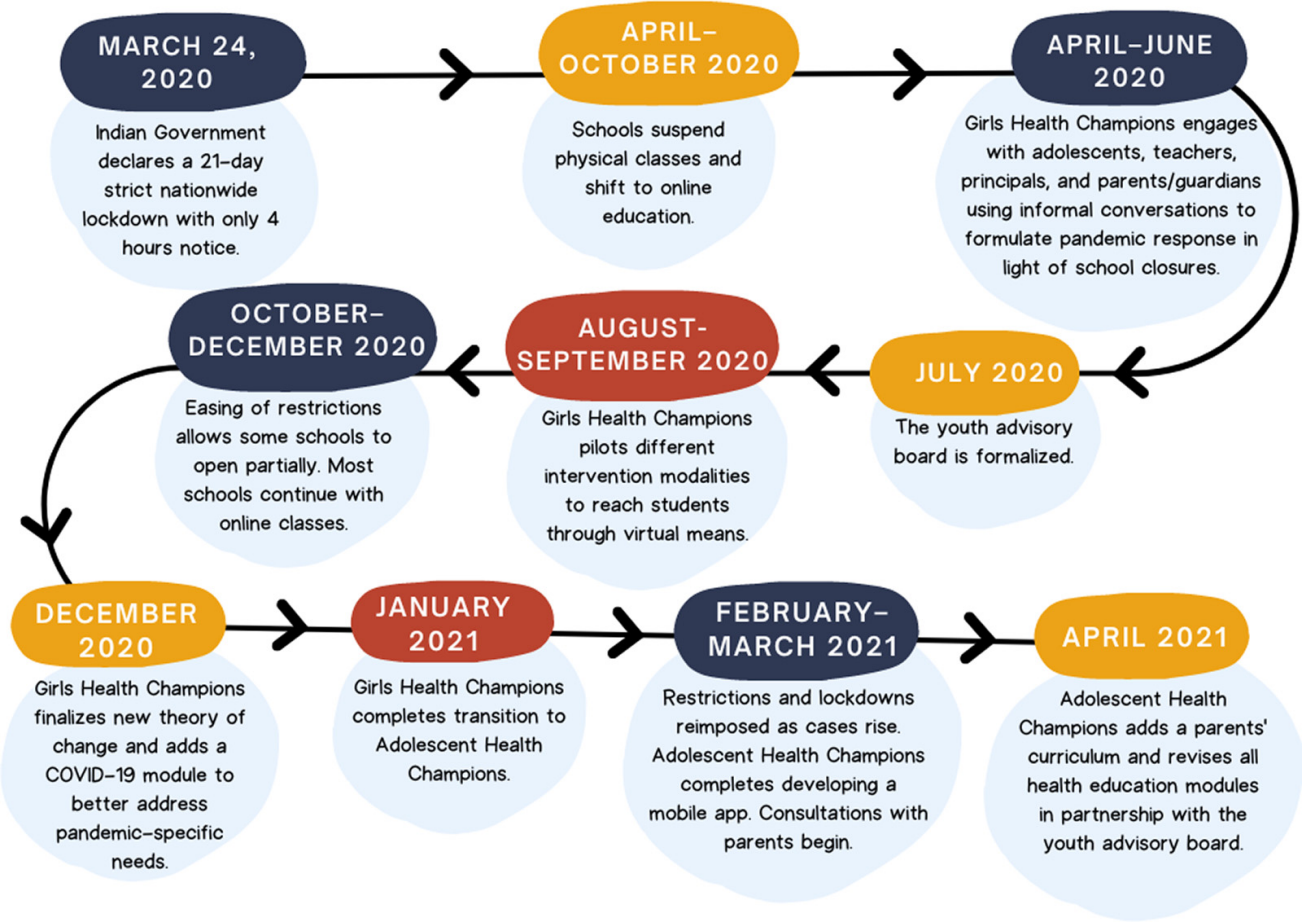
Timeline of Responsive Feedback Implementation by the Adolescent Health Champions Program

In the early stages of the pandemic (April–June 2020), AHC began undertaking informal conversations with adolescents, teachers, principals, and parents/guardians to understand the unique needs of AHC's adolescent participants and inform any pandemic-related response by AHC. We describe the valuable community perspectives collected through these consultations, how these consultations were formally integrated into the working of AHC through the creation of a YAB, and how consultations with the YAB proved integral to the development of a cohesive and meaningful response to address pandemic-related challenges for adolescents (Figure 2 shows the timeline of RF implementation).

## USE OF RF IN THE YAB DEVELOPMENT PROCESS

### Informal Consultations With Adolescents (April–June 2020)

School closures resulted in a new paradigm for the AHC team, as in-person peer education sessions were no longer possible. As a result, the AHC team contacted stakeholders—including adolescents, teachers, principals, and parents/guardians—and collected feedback through calls, emails, and WhatsApp messages to better understand how to navigate the unprecedented challenges posed by the pandemic.

AHC contacted champions through school-teachers and principals and with the consent of their parents/guardians. At this stage of informal consultation, power dynamics between the AHC team and the champions were, at best, mitigated by the strong rapport established during training and then further reduced through ongoing conversations and consultations over the course of a year. To address power dynamics more systematically and institutionalize these consultations into the working of AHC, the decision was made to formally institute a YAB.

### Formal Institution of the YAB (July 2020)

In June 2020, the AHC team detailed the time commitment and envisioned role for YAB members in an interest form that was sent to all adolescents who had acted as champions during previous programming in Mumbai. This form was sent on joint WhatsApp groups where teachers, parents/guardians, and students of partner schools were present. The pool of partner schools included 3 schools located in central, eastern, and northeastern Mumbai. School 1 was an all-girls, religious minority private school located in a majority Muslim community in central Mumbai. School 2 was a coed, private school in eastern Mumbai run by a large corporation and attended by many children of workers at the corporation's factory. School 3 was a coed, government-aided private school in northeastern Mumbai, with the majority of students from families where parents/guardians were daily wage earners. In all 3 schools, English was the language of instruction for all classes, and classes were held during the day (as opposed to in the evenings).

In July 2020, 8 adolescents volunteered to participate as YAB members. Of these, 2 identified as boys and 6 identified as girls. In February 2021, AHC received interest from 13 more champions from 7 newer intervention schools, bringing the total YAB membership to 21 adolescents aged 13–16 years from 10 schools, of which 6 identified as boys and 15 as girls.

The inclusion criteria applied by the AHC team (adult team members) when seeking YAB members were: (1) previous participation as a peer educator in the AHC program, (2) parent/guardian consent, and (3) adolescent assent. Given that all 21 adolescents who volunteered to join the YAB met these criteria, all were included in the YAB upon expression of interest. Before formally engaging the YAB, the AHC team asked for interest from participants to serve as YAB cochairs. Two young people (who are also coauthors of this article) shared an interest in this role and worked with adult team members to create weekly agendas and lead forthcoming YAB meetings. All adolescents received a token of appreciation (notebook and pen), certificate, t-shirt, and letter of recommendation and were part of multiple felicitation ceremonies recognizing their participation.

In terms of representativeness of the YAB members, the authors consider the YAB to be closely representative of the adolescent demographic that: (1) lives in urban areas, (2) can afford to attend English-medium private and government-aided schools, (3) has access to phones, computers, and the Internet. Data on income levels, wealth quintiles, and caste were not collected. However, 2 of 10 schools were located in lower-income communities in Mumbai, and close to 40% of YAB participants belonged to minority religious communities. The formal and informal engagement with YAB members was conducted in English. The ability to speak English is an aspirational goal and a marker of material, social, and cultural capital in India, therefore suggesting some degree of privilege.^14^ We recognize that the sample of YAB members represents a subset of urban adolescents in Mumbai, specifically, adolescents who are able to regularly access smartphones and the Internet and who attend English medium schools but who also represent a range of socioeconomic and religious backgrounds. As such, the findings cannot be generalizable to all urban adolescents or to adolescents living in rural parts of India.

### Engagement With the YAB (July 2020 Onward)

As part of the formal functioning of the YAB, the AHC team focused on engaging in consultation, ideation, consensus building, cocreation, and codesign processes with YAB members. Virtual bimonthly meetings were held over Zoom, lasting for approximately 1 hour, and were attended by YAB members and the AHC team. The agenda for these meetings was jointly set by YAB cochairs and the AHC team and would revolve around different thematic areas of work, such as curricular content and school programming. During the initial meetings, YAB members, facilitated by the YAB cochairs, helped the YAB create a vision document that served as the basis for forthcoming meetings. This vision document was completed by October 2020 and later revised in June 2021. The document included: (1) group values, (2) purpose, (3) focus areas, (4) recruitment criteria, (5) term limits, and (6) recognition for YAB member participation.

As part of the formal functioning of the YAB, the AHC team focused on engaging in consultation, ideation, consensus building, cocreation, and codesign processes with YAB members.

Additionally, individual check-ins were conducted every 6 months with each YAB member, ensuring that all members were given opportunities to learn, grow, and share their goals. Before each check-in, YAB members received a survey that enabled them to share 3 personal goals and 3 professional goals, which the AHC team would review and provide support toward. Further, anonymous polls were used roughly every 3 months to elicit feedback on the perceived purpose, strengths, challenges, and impact of the YAB. These polls helped ensure that each YAB member could express their opinions independently of any group consensus or pressure. Poll results were accessed by the AHC team, shared with the YAB, and used to drive upcoming discussions. An example of a poll question is: “Do you feel that our organization's name should be Girls Health Champions or Adolescent Health Champions?”

#### Design Thinking Workshops

**FIGURE 3:**
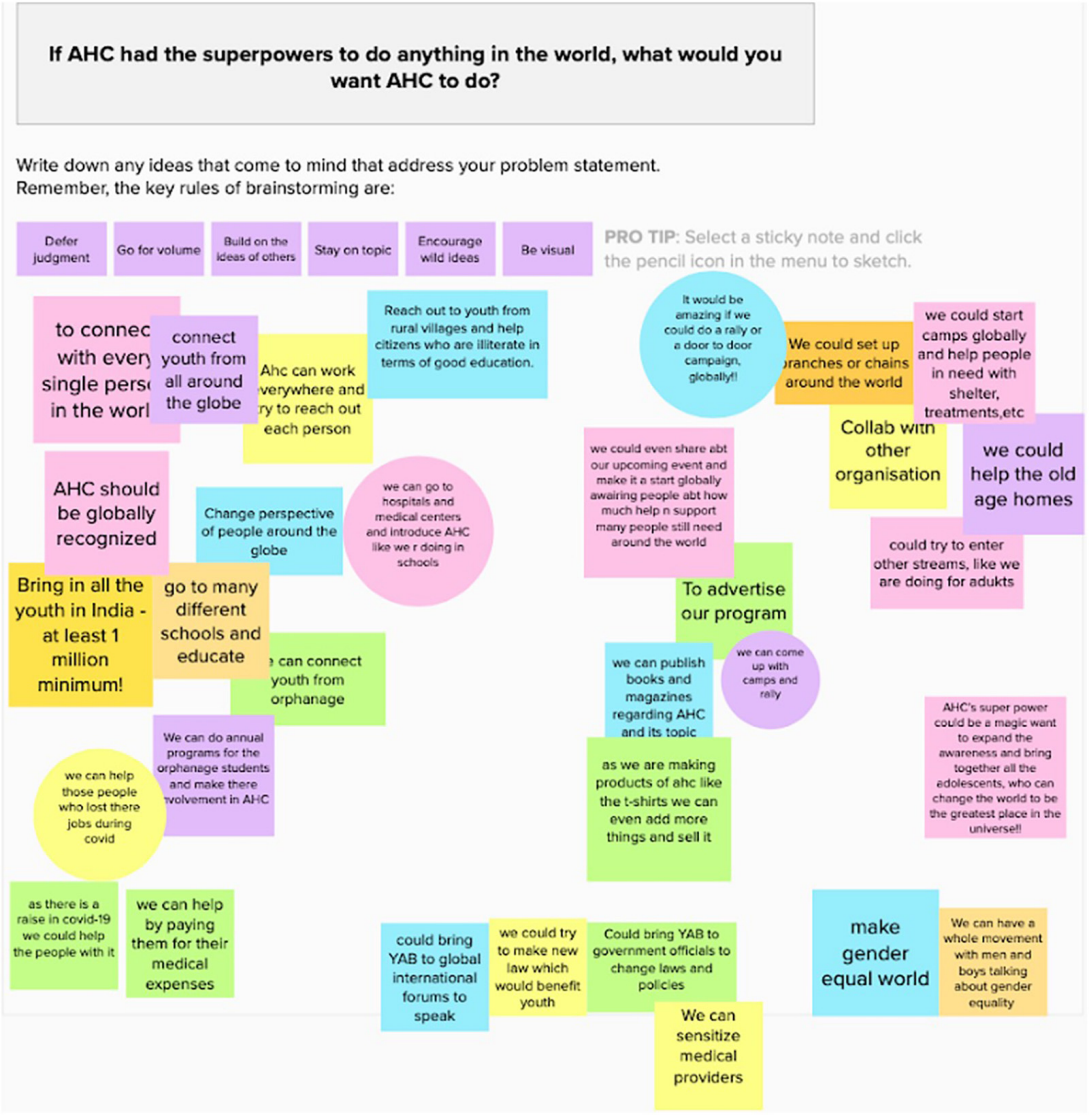
Digital Whiteboard From Design Thinking Workshop Held by the Adolescent Health Champions Program^a^ Abbreviation: AHC, Adolescent Health Champions. ^a^ Workshop held in January 2021.

Design thinking was a crucial method used by the AHC team as part of its RF approach. The monthly workshops were led by YAB cochairs with the AHC team and trained young adults and became a way to ensure that AHC was taking action based on the areas adolescents felt were most important to address. These workshops also enabled AHC to build a culture of innovation and experimentation. Design thinking served as a “systematic process for linking ongoing implementation learning to modifications in project design” via a sustainable “feedback loop” system.^15^ Studies demonstrate design thinking generates “solutions that are constantly oriented toward the needs of users.”^16^ In each monthly workshop, YAB and AHC team members would bring to light major challenges and problems faced by either the AHC team or adolescents. The AHC team used design thinking as a way for all team members (adolescents, volunteers from across disciplines including medicine and public health, and educators) to work together to address these challenges. Design thinking workshops involved using an online multimedia platform (Mural or Miro, combined with Zoom) to help solve a pertinent challenge faced by the YAB members, AHC participants, or by AHC at the organizational level. To generate a broad scope of solutions, during sessions, all participating adolescents or team members individually posted their proposed ideas as sticky notes or images on the platform. The facilitator organized these inputs by emergent themes, after which a few spokespersons discussed key findings. Once themes were categorized, participants had an opportunity to vote on categories and proposed solutions. Trained design thinking experts (such as 1 of the coauthors) were particularly helpful in training and guiding our organization to incorporate and use this technique. We also used silent brainstorming sessions, breakout rooms, and a digital whiteboard (for those who could not access the online multimedia platforms). These digital whiteboards also enabled design thinking sessions to include more objective measures of expressing opinions, such as ratings or icons. Figure 3 provides an illustrative example of a design thinking workshop.

## OUTCOMES OF THE AHC RF APPROACH

Using the various methods previously described, the AHC team and YAB together were able to: (1) understand pandemic-specific challenges faced by adolescents; (2) substantially revise our theory of change (TOC); (3) pilot new modes of intervention delivery; (4) design a curriculum for parent/guardians and a new COVID-19 module; (5) create an AHC mobile app prototype; (6) develop a new, more gender-inclusive name and visual identity for AHC; (7) change the overall structure, adolescent-friendly nature, and agility of AHC; and (8) help clarify the future directions taken by AHC.

### Adapting Programming to Adolescent Needs

YAB members shared the significant challenges posed by the pandemic, including social isolation and impacts on their physical and mental health. Engaging with YAB members further brought to light contextual features of adolescent experiences and how best to proceed during the pandemic (Table 2). YAB members provided important guidance and insider feedback on the status of their schooling, social and home environments, and the areas in which the AHC team could intervene. Based on this feedback, AHC then shifted focus to modifying programming.

**TABLE 2.** Scoping the Challenges: Agile Feedback from Youth Advisory Board Members

Profile	Theme	Insights
YAB adolescent and coauthor on this article from minority school	Education, social support	Key insight: Lack of peer connection and social support. Illustrative quote: “As the schools are closed due to COVID-19, the education of the children and adolescents has undergone a great change. Schools have started virtual lectures for students for the very first time. This reduces the socializing of the students.”
YAB adolescent and coauthor on this article from minority school	Education, Internet connectivity challenges	Key insight: Difficulty navigating online schools with Internet connectivity issues. Illustrative quote: “… if I talk about online classes, teachers are at their best, but there are students who are unable to join, especially in rural areas of India because of the Internet. The situation is very threatening.”
Adolescent participant in AHC school programming	AHC programming	Key insights: Programs should be adapted to different modalities based on each school's needs and the connectivity of its students; virtual, hybrid, asynchronous, and in-person delivery should be used when appropriate.

Abbreviations: AHC, Adolescent Health Champions; YAB, youth advisory board.

Engaging with YAB members further brought to light contextual features of adolescent experiences and how best to proceed during the pandemic.

### Experimenting With New Intervention Delivery Modes

YAB members emphasized the importance of continuing the program given the significant physical and mental challenges adolescents were experiencing during the pandemic, but they expressed that the AHC program needed to remain agile and adapt to the changing academic and social environment (Table 3). Based on these insights, AHC piloted these various modalities in different schools in collaboration with the YAB and students and teachers from these schools.

**TABLE 3.** Youth Advisory Board Feedback on Adapting Adolescent Health Champions Program Delivery Modes During the Pandemic

Program Modality	School Characteristics	Program Approach
Fully virtual programming	Majority of students have access to Internet; ongoing virtual classes	Adolescents lead the virtual intervention in their schools by recruiting new champions, teaching newly selected peer educators the content, coordinating with school administrators to select times and dates for the program, and subsequently working with other peer educators to teach the material.
Hybrid programming	Limited and unreliable Internet connectivity; limited online classes	One adolescent or school staff who has access to technology would use it to connect to the AHC team and subsequently allow the AHC team to share the intervention virtually with adolescents on school premises.
Asynchronous programming	Poor and erratic Internet connectivity; little to no online class programming	Adolescent leaders encouraged the AHC team to create video content that could be streamed by adolescents on their own time and utilize WhatsApp to disseminate AHC curricular content.

Abbreviation: AHC, Adolescent Health Champions.

YAB members continued to share feedback regarding areas for growth, particularly curricular content. YAB members specifically highlighted the need to: (1) incorporate parents/guardians as stakeholders in the AHC program, (2) incorporate new content relating to the COVID-19 pandemic, and (3) leverage technology to provide an additional layer of ongoing support to participating adolescents during the pandemic, all of which became important and distinct work streams for AHC in the subsequent months (Table 4).

**TABLE 4.** Iterative Youth Advisory Board Feedback on Building Out Adolescent Health Champions Programming

Stakeholders	Themes and Insights	Output
Adolescents	Need for content and session for parents/guardians given proximity between adolescents and parents/guardians during the lockdown and the potentially sensitive nature of content covered by AHC, such as sexual and reproductive health. Adolescents shared concerns about incorporating parents/guardians directly into adolescent-led peer education sessions; many suggested creating separate sessions for parents/guardians instead.	Significant involvement of adolescents (led by 2 YAB members) in developing parent/guardian curriculum to sensitively incorporate adolescent perspectives while maintaining a separate space for adolescent participants to continue their own peer education sessions.
Parents and guardians	Uncertainty about how to best support adolescents during the pandemic and a lack of awareness about topics covered in the AHC curriculum.	Incorporated sessions for parents/guardians using new curriculum that includes excerpts from the AHC curriculum and covers themes like autonomy vs. independence, fear vs. love, and positive communication with adolescents.
Adolescents	Need for reliable and accurate COVID-19 information, with many adolescents expressing they were unable to find reliable information on their own.	Creation of new COVID-19 module (Figure 6) by AHC and YAB covering information related to physiology, signs and symptoms, diagnosis and management, impacts on marginalized populations, where to seek help, and how adolescents can manage mental health during the pandemic.
Adolescents	Creation of AHC mobile app could facilitate wide dissemination of adolescent content and augment the virtual peer education in their schools given that adolescents are familiar and comfortable with mobile technology and meet health-related needs.	Over 10 months, YAB members codesigned an AHC mobile app prototype (Figure 7); they chose visuals, provided inputs on user flow, recommended ways to protect adolescent privacy and safety, vetted the usefulness of resources for seeking physical and mental health support, suggested gamification features, and vetted app-friendly content. Several YAB members participated in (1) beta testing sessions as the app was undergoing development and bug testing; (2) focus group discussions to share specific feedback on different sections of the app; and (3) brainstorming sessions on launching and rolling out the app to students in their respective schools, addressing issues such as optimal timing, phased rollouts, and bandwidth concerns. The app is currently being pilot tested.

Abbreviations: AHC, Adolescent Health Champions; YAB, youth advisory board.

### Refining the TOC

**FIGURE 4:**
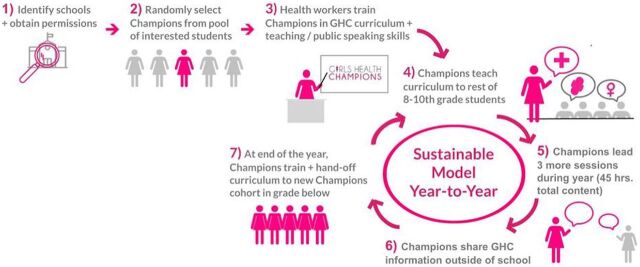
Pre-Pandemic Theory of Change for the Girls Health Champions Program Abbreviation: GHC, Girls Health Champions.

**FIGURE 5:**
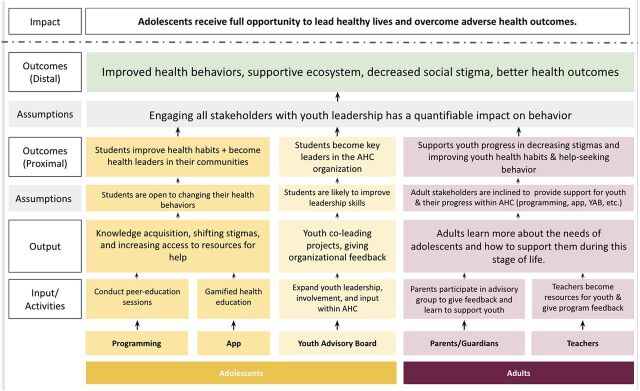
Responsive Feedback-Informed Theory of Change for the Adolescent Health Champions Program Abbreviations: AHC, Adolescent Health Champions; YAB, youth advisory board.

In the early stages of the YAB, adolescent members also worked with the AHC team to refine our TOC to provide structure to the YAB and AHC as a whole (Figure 4). This TOC evolved during the pandemic (Figure 5), with key elements being added in response to YAB insights including: (1) continuing to conduct peer education sessions (virtually or in person as possible during the pandemic) in the immediate term and expanding to new areas with limited Internet connectivity that may have had worse challenges during the pandemic, including rural communities in the long-term post-pandemic; (2) creating a mobile health app for adolescents across India; (3) encouraging parents/guardians and teachers to participate in their child's or student's adolescent health education and conducting additional programming with such stakeholders; and (4) allowing for upward mobilization and leadership opportunities for adolescents within AHC as an organization. Further, YAB members suggested that additional goals of AHC programming should include improving health behaviors, building a supportive health ecosystem for adolescents to ultimately improve adolescent health outcomes, and evaluating the impact of the program.

### Designing New Content for Parents/Guardians and on COVID-19

YAB members highlighted the importance of (1) incorporating parents/guardians as stakeholders in the AHC program and (2) incorporating new content relating to the COVID-19 pandemic. As such, it was decided that the AHC team and the YAB would codesign a parent/guardian curriculum (to be shared with parents/guardians) and a COVID-19 module (to be delivered as the fifth module in the AHC health education curriculum). These 2 new pieces of content were developed over 2 months. The AHC team observed that YAB members took significant ownership in designing this content and structuring the associated sessions. For example, during the development of the COVID-19 module, YAB members pointed out the need to add content on additional support in dealing with the impacts of the pandemic on their day-to-day life (such as school closures), understanding the broader social impacts of the pandemic, and coping with mental health impacts. This led to augmenting content related to mental health and social support in the final versions of the module.

For the curriculum for parents/guardians, the AHC team and YAB involved 2 parents/guardians from stakeholder schools who shared core experiences and important elements that could be added into the curriculum. The curriculum further developed over the course of an additional 2 months with the YAB, with sessions using polls and design thinking to foster consensus around including content on communication strategies, healthy relationships, and AHC as a whole and its peer education model and adolescent health focus. Based on the RF process, AHC began all subsequent programming in schools by first conducting a session with parents/guardians—leveraging this newly created curriculum—to support them with their unique needs as caretakers of adolescents during the pandemic and to facilitate their buy-in with their child's participation in AHC programming at school.

### Developing an AHC Mobile App

**FIGURE 6:**
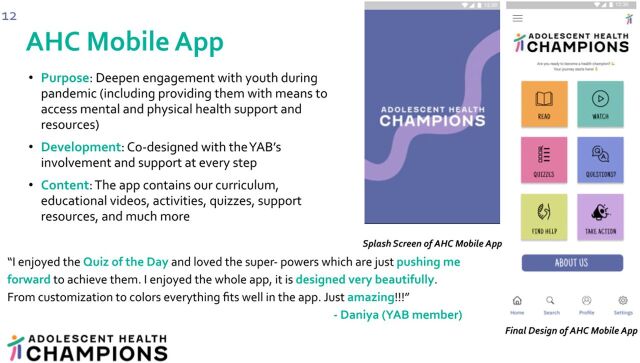
Adolescent Health Champions Mobile App Iteratively Codesigned With the Youth Advisory Board Abbreviations: AHC, Adolescent Health Champions; YAB, youth advisory board.

**FIGURE 7:**
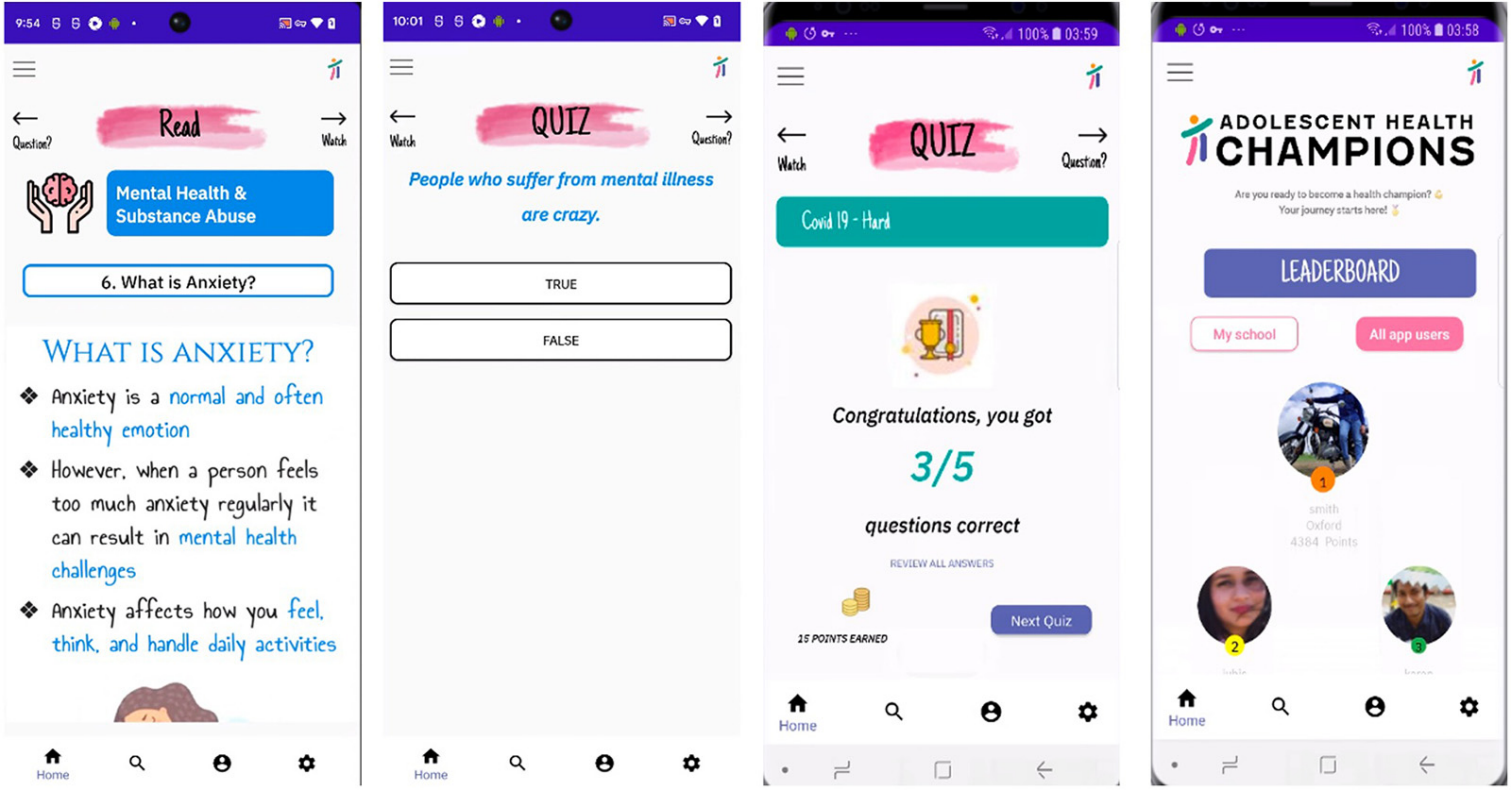
Screenshots From the Adolescent Health Champions Mobile App

The creation of the AHC mobile app and its addition to AHC's TOC was a direct consequence of the feedback and engagement of YAB members (Figure 6). YAB members emphasized the need to create mobile technology for adolescent health, as they believed a mobile app would enable them to (1) deepen their learning around adolescent health, (2) access content and clinical support resources in the future as needed, (3) disseminate health information to others around them, and (4) better stay connected with AHC. YAB members further cited the growing use and comfort with mobile technology among adolescents and widespread access to smartphones as reasons for the AHC team to invest in a mobile app as part of AHC's pandemic response. Over the course of 10 months, YAB members codesigned the prototype with AHC team members and an external developer, with all parties using design thinking to formulate key components of the app (Figure 7). The YAB's role included providing guidance on (1) the apps' user interface design and user experience design, (2) ways to maintain adolescents' privacy and safety, (3) vetting resources where adolescents could seek physical and mental health support, (4) gamification features, and (5) health content. AHC's mobile app is currently being pilot tested in several AHC partner schools, and the YAB was instrumental in creating a prototype that AHC plans to continue building out in the coming years.

The creation of the AHC mobile app and its addition to AHC's TOC was a direct consequence of the feedback and engagement of YAB members.

### Changing From Girls Health Champions to Adolescent Health Champions

One of the most important outcomes of the RF approach was the change of the organizational name from Girls Health Champions to Adolescent Health Champions. During a YAB meeting, multiple female-identifying adolescents expressed the importance of having a more gender-inclusive umbrella and organizational name to foster allyship across all genders (i.e., to include male-identifying adolescents and adolescents whose gender identity and presentation do not easily fit a strict male-female binary conception of gender) and drive systemic change. These suggestions were viewed positively by the AHC team and welcomed as an opportunity to reflect on the organization's positioning and branding. A subsequent design thinking workshop enabled the AHC team to reflect on the benefits (gender-inclusivity and involving all genders in the movement for gender justice) and the challenges (no longer having an overt focus or emphasis on girls) of shifting external branding to AHC. Ultimately, the workshops and the ability to vote on the decision collectively enabled the AHC team and YAB to change direction using a consensus-building approach. Several studies suggest the importance of involving adolescents representing all forms of gender identity and expression in interventions seeking to address gender inequalities and unequal gender norms.^17^^,^^18^ Thus, continued adolescent engagement and leadership helped AHC as an organization in shifting to a more gender-inclusive name and visual identity, which better positioned AHC to improve adolescent health outcomes and gender inequality while also aligning with stakeholder feedback.

After deciding to shift from Girls Health Champions to AHC, the team went through a 3-month process involving multiple iterations of design thinking with an external branding firm to recreate AHC's visual identity. Team members were asked to build mood boards to visually represent AHC and to write keywords to describe the organization. At the end of the process of transitioning AHC's visual identity, all YAB and team members were asked in an anonymous survey to share if they preferred the organization's old or new identity, and there was unanimous positive feedback in support of the new AHC identity and branding.

An anonymous survey of all YAB and AHC team members yielded unanimous positive feedback in support of the new AHC identity and branding.

## DISCUSSION

RF—via design thinking, pilot testing, and iterating—led to changes in AHC's organizational approach and facilitated continued organizational adaptation and growth. AHC convened stakeholders by creating a YAB, seeking their feedback and inputs through surveys, individual check-ins, and design thinking workshops. AHC subsequently tested different modalities for the peer education intervention and considered new possibilities for AHC's work such as including developing new curricular content, creating a mobile app, transitioning the organization from Girls Health Champions to AHC, and meaningfully incorporating adolescents into AHC's leadership structure.

While AHC originally had a goal of reaching 10,000+ adolescents in 2020 through its in-person peer education model, the YAB's presence and perspective enabled the AHC team to recognize that adapting to a changing environment based on necessity, stakeholder feedback, and the organization's TOC was more important than meeting the organization's pre-pandemic target. Viswanath et al. similarly point to the importance of TOCs in ensuring interventions focus on overarching goals rather than “becoming fixated on what the program is currently doing.”^13^ While AHC was not able to meet its intended 1-year goal of reaching 10,000+ adolescents in the 2020–2021 academic year, through these various new modalities of intervention, the AHC team and YAB—by overhauling AHC's TOC and adapting and augmenting the organization's vision—were able to maintain the organization's focus on peer education while also educating over 4,000 adolescents virtually that year.

Regarding the AHC mobile app, AHC program participants had suggested (through post-intervention qualitative and quantitative surveys and focus group discussions conducted before the pandemic) that AHC incorporate a technology-based solution into its programming. Before the COVID-19 pandemic, AHC had held a contest in several partner schools in November 2019, during which adolescents pitched their ideas for a hypothetical AHC mobile app. However, the AHC team had been apprehensive about moving forward with the mobile app, as it remained focused on its traditional in-person intervention. Pandemic uncertainty, the creation of the YAB, design thinking sessions, polls, the resounding interest in this modality of intervention from adolescents themselves, and the RF approach enabled the AHC team to be nimble and explore new possibilities. Further, the opportunity to codesign and iterate on the creation of an AHC mobile app in a more hands-on manner with adolescent members made the AHC team much more confident that an app could address adolescents' needs, be comprehensible and useful for them, and ultimately be a successful intervention model.

Similarly, the creation of the curriculum for parents/guardians was an important milestone for AHC, as it was the first time AHC conducted sessions with adults. As the AHC team started conducting the sessions, it recognized that many parents/guardians also required significant support and guidance about how to best interact and maintain healthy relationships with children, particularly given the social and mental health impacts of the pandemic on adolescents. By involving adolescents in the design process and seeking feedback from them and their parents/guardians after each session, AHC was able to design a curriculum for adults that sensitively incorporated adolescent and parent/guardian voices and current realities of the pandemic while maintaining a separate space for adolescent participants to continue their own independent peer education and learning sessions. Such a curriculum was also instrumental in augmenting stakeholder buy-in and understanding of the AHC program as the curriculum was shared with guardians before the AHC intervention was conducted in schools and served as a space for parents/guardians to learn about the program and ask questions.

Importantly, the incorporation of stakeholder involvement in AHC through adolescent leaders has fundamentally restructured notions within the organization surrounding expertise in the field of adolescent health, as well as in specific programmatic areas such as research, marketing, technology, partnerships, and human resources. As described, YAB expertise was important in shaping the vision and direction of the organization. AHC has since shifted from more of a vertical leadership structure—in which those traditionally considered experts in adolescent health (physicians, educators, and organizational leadership) or programmatic areas set the vision and executed it—to a flatter, more horizontal structure in the organization's leadership and team that involves adolescents in all aspects of decision-making and promotes their acquisition of skills in programmatic areas. After spending 1 year during the pandemic on the YAB, 6 members in the 10th and 11th standards expressed an interest in joining the AHC core team and officially joined as volunteer team members in June 2021. Many of these adolescents (some of whom are coauthors and helped brainstorm, outline, develop, write, and edit this article) have co-led or co-coordinated verticals, including mobile app development and human resources. In the future, once these adolescents are aged 18 years, the authors envision them leading across every part of AHC, including joining AHC's Board of Directors.

Additionally, the AHC team has made a conscientious effort to incorporate adolescents into its research and evaluation efforts. In AHC's experience, incorporating adolescent stakeholders in the evaluation process has enabled the organization to immediately act on findings in real time and allowed adolescents to develop their own skill sets. Two coauthors of this article (including 1 YAB member) coauthored an article on the importance of youth-led interventions in *The Lancet Child & Adolescent Health*,^19^ and the coauthorship of this article on RF with adolescents represents another example of how AHC has worked to shift hierarchies and paradigms both internally as an organization and in its external messaging, positioning, and publications since the pandemic. All YAB members were given the opportunity to become coauthors of this article, and after the AHC team explained to them the requirements for participation (attending additional meetings, engaging in discussions, providing feedback, writing sessions, etc.), all those who volunteered were selected as coauthors. Ultimately, the RF approach enabled new possibilities to pause and reflect on hierarchies in AHC, including surrounding age. Having the YAB and YAB coleaders guide discussions served as a means of destabilizing traditional power structures, which resulted in a more enabling environment where adolescents could more comfortably contribute their opinions and see their ideas come to fruition. Additionally, the use of multiple platforms—from anonymous polls and surveys to check-ins—resulted in adolescents developing a deeper connection to the YAB and the AHC team, as well as feeling more comfortable providing honest feedback about their experiences within YAB meetings.

Incorporating adolescent stakeholders in the evaluation process has enabled AHC to immediately act on findings in real-time and allowed adolescents to develop their own skill sets.

Another important consequence of using a community-partnered RF approach has been a shift in how the AHC team measures organizational success, from a predominant focus on traditional metrics, such as the number of adolescents educated or peer educators trained, to a broader, more holistic vision of success centered on innovation; adolescent inclusivity in all aspects of the organization (including its name) and decision-making; and AHC's ability to collectively iterate, resolve challenges, and adapt to change. To cement this culture into the organization, an AHC young adult team member proposed creating an AHC vertical (a specialized and distinct area of work and responsibility) dedicated specifically to innovation to facilitate ongoing ideation and experimentation.

### Challenges of Following an RF Approach

A significant challenge with undertaking an RF approach is that it may slow down the overall process of creation and execution of an intervention, especially in the setting of a small team. As can be seen in AHC's own pandemic response, pausing to take in stakeholder feedback did require the organization to often spend 2–4 months to synthesize feedback across issues, as with the creation of the curriculum for parents/guardians and the COVID-19 module, pilot testing programming through different new modalities, and developing the AHC mobile app. This also posed a challenge for meeting prior proposed deadlines or metrics of success. RF and engaging multiple stakeholders can also make it difficult to gain consensus while staying true to multiple competing opinions. Finally, using approaches such as design thinking can prove challenging in terms of authentically and representatively engaging young people, especially when sessions are led by adult leaders.

To mitigate this potential challenge, AHC consistently used anonymous feedback tools, such as surveys and polls, to allow everyone the opportunity to share their ideas independently. Furthermore, the AHC team leveraged consensus-building activities like design thinking workshops and ensured, as much as possible, that these sessions were led by trained adolescent and young adult facilitators to encourage collaboration, group brainstorming, mitigate power differentials within the group, and encourage adolescent participation. Such methods have enabled AHC to synthesize different ideas, aggregate and understand majority perspectives and opinions, and build organizational consensus. Pausing, reflecting, and iterating were also beneficial for maintaining the participation and engagement of AHC team members, developing AHC's organizational structure and culture, and modifying AHC's overall intervention into a more holistic, multipronged program (including virtual and hybrid peer education, a mobile app, and new curricular content, among other aspects), rather than a single, rigid intervention (i.e., an in-person peer education intervention).

### Expanding AHC's Programming and Future Directions

In the YAB vision document, adolescents from Mumbai shared the critical importance of expanding the program and adapting it to rural communities in India, as well as continuing to evaluate the impact of AHC's programming. Since the end of the acute lockdown of the COVID-19 pandemic, AHC has partnered with 2 community-based NGOs—URMUL Setu in rural Rajasthan and Ugam Education Foundation in Jharkhand (in coordination with the Bokaro District Administration, Government of Jharkhand)—to expand the program to more than 4,000 adolescents in rural India. AHC codeveloped a context-sensitive curriculum in conjunction with community stakeholders using a similar codesign and joint knowledge creation process that the organization first learned through its experiences with the Mumbai-based YAB. Finally, an RF approach remains an integral part of AHC's ongoing efforts in rural India, as AHC has tested using both virtual and in-person modalities of education where possible and continues to be open to changing curricular content and evaluation methods based on stakeholder feedback and pilot findings. Importantly, AHC has involved a YAB and community advisory board in its efforts in both Rajasthan and Jharkhand. Moreover, AHC has included options for Hindi language participation and continues to use tools, such as anonymous feedback, surveys, polls, and design thinking activities, with trained facilitators. Lastly, given the Mumbai-based YAB's advocacy for further understanding the role AHC's programs may play in influencing behavioral change in adolescents, AHC's forthcoming research has involved evaluating its peer education model in rural Jharkhand and Rajasthan with a set of control schools.

### Limitations

There are several notable limitations of this case study. First, YAB members in this case study were drawn from a subset of adolescents who had already been trained and supported as peer educators, were enrolled in private and government-aided schools, and had the means to participate in virtual convenings where discussions were held in English. As such, the members who were involved in this case study held a degree of privilege (geographical, educational, social, and economic) that limits the overall generalizability of the acceptability and efficacy of employing the RF approach in conjunction with YABs in other settings. Of note, since August 2022, AHC has worked closely with YAB members from historically marginalized communities in rural Jharkhand and Rajasthan, and AHC's insights from these experiences will be the basis of forthcoming publications.

Second, AHC as an organization has been funded by several small grants, including the Harvard T.H. Chan School of Public Health, WeWork Creator Awards, and the D-Prize, all of which provided unrestricted grant funding for its interventions. Such unrestricted funding could be a reason for the effectiveness and acceptability of the RF approach in the context of the AHC team and programming. Donors could be hesitant to fund investments in collaborative stakeholder engagement mechanisms, such as YABs, as doing so could shift the attention of organizations away from achieving numerical targets and result in programmatic pauses, given that such shifts and pauses may be required to successfully use the RF approach. As such, some outside donors may be unsupportive of an RF approach, and this may serve as a barrier for organizations globally seeking to experiment beyond their benchmarked goals.

Finally, certain features of AHC have enabled a spirit of iteration within the organization, including its young team, with 80% of members aged younger than 25 years; being in existence and operating less than 10 years; and having less than 20 team members. Some studies suggest that youth-led organizations are more open to novel ideas.^14^ Additionally, having a younger team (in terms of age range of team members) might have enabled adolescents to engage more freely and may have mitigated power dynamics often seen in adolescent-serving organizations involving a vast majority of older adults. Smaller organizations similarly have been shown to be more intimate, providing team members the opportunities to more actively share feedback, raise challenges, and engage effectively.^15^ These features may limit the broad applicability of these findings to other organizations.

## CONCLUSION

The creation of the YAB and use of the RF approach have had significant positive impacts on AHC as an organization and have been instrumental in its growth and development, as well as its pandemic response. Having actively used the RF method over the last 2 years, AHC can strongly attest to the overall strength of the approach in developing programming and that its benefits far outweigh its challenges. The centering of adolescent voices through the incorporation of a YAB in AHC has further enabled AHC to respond more effectively to adolescent needs during the pandemic with robust community and parent/guardian support. Such support continues to lay the foundation for AHC's growth and impact throughout the course of the pandemic and beyond. We hope this article serves as an example of the possibilities for organizational adaptation using the RF approach with youth and might inspire other youth- and community-serving organizations to actively use such an approach and meaningfully incorporate YABs in all aspects of their interventions.

## Notes

**Peer Reviewed**

**First published online:** October 6, 2023.
